# Hematological Parameters for Predicting Mortality in Acute Exacerbation of Chronic Obstructive Pulmonary Disease

**DOI:** 10.3390/jcm12134227

**Published:** 2023-06-23

**Authors:** Rohan Karkra, Chaya Sindaghatta Krishnarao, Jayaraj Biligere Siddaiah, Mahesh Padukudru Anand

**Affiliations:** JSS Medical College and Hospital, JSSAHER, Mysuru 570015, Karnataka, India

**Keywords:** AECOPD, mortality, predictor, hematological markers, neutrophil lymphocyte ratio

## Abstract

(1) Introduction: COPD is a common and serious condition affecting a significant proportion of the population globally. Patients often suffer from exacerbations which lead to the worsening of their health status and respiratory function, and can often lead to death. Quick and cheap investigations are required that are capable of predicting mortality in patients with acute exacerbations that can be applied in low resource settings. (2) Materials and methods: This was a retrospective study carried out using hospital records of patients admitted for AECOPD from 1 January 2017 to 30 November 2022. Chi-square test (for sex) and Student’s *t*-test were used to look for significant associations. Receiver Operating Characteristics (ROC) curves were plotted and Area Under Curve (AUC) values were calculated for various hematological parameters. Youden’s J was used to identify the ideal cut-off with optimal sensitivity and specificity. Multivariate Cox regression was used to identify independent hematological predictors of mortality. Kaplan–Meir survival plots for neutrophil lymphocyte ratio (NLR) with the optimal cut-off were plotted. (3) Results: Amongst the 500 patients, 42 died while 458 survived, giving a mortality rate of 8.4%. NLR had the strongest association with mortality. The cut-off for various parameters were: NLR 14.83 (AUC 0.73), total leukocyte count (TLC) 13,640 cells/mm^3^ (AUC 0.60), absolute neutrophil count (ANC) 12,556 cells/mm^3^ (AUC 0.62), derived NLR (dNLR) 9.989 (AUC 0.73), hemoglobin 11.8 mg/dL (AUC 0.59), packed cell volume (PCV) 36.6% (AUC 0.60), and platelet lymphocyte ratio (PLR) 451.32 (AUC 0.55). (4) Conclusions: In patients with acute exacerbation of COPD, NLR was strongly associated with mortality, followed by dNLR. Cox regression identified NLR as an independent predictor of mortality.

## 1. Introduction

Chronic Obstructive Pulmonary Disease (COPD) is a common and debilitating condition affecting the respiratory system. It creates a huge social, economic and psychological burden on society, as well as physical distress. The etiology is diverse, complex, and multifactorial, and gene–environment interactions have been linked to its development [1]. It is officially defined as a heterogeneous lung condition characterized by chronic respiratory symptoms (dyspnea, cough, sputum production, exacerbations) due to abnormalities of the airways (bronchitis, bronchiolitis) and/or alveoli (emphysema) that cause persistent, often progressive, airflow obstruction [1]. Patients with COPD present with complaints such as productive cough, breathlessness, chest tightness, and easy fatiguability, and over time, patients may develop serious complications such as cor pulmonale, pneumonia, anxiety, depression, and emaciation, ultimately resulting in death. In its latest 2023 report, the Global Initiative for Chronic Obstructive Lung Disease (GOLD) identified COPD as one of the top three causes of mortality globally [1]. A Burden of Obstructive Lung Disease (BOLD) report observed an overall prevalence of 11.8% among males and 8.5% among females [2]. It is estimated that 3 million people die of COPD every year [3].

A common cause for patients with COPD to present to the hospital is an acute exacerbation of COPD (AECOPD). Exacerbation is defined as an acute worsening of symptoms of dyspnea, cough, and sputum production from the patient’s baseline [4]. These exacerbations can be triggered by a viral illness or a spike in ambient air pollution [5]. This may lead to hospitalization, worsening of lung reserve, lung failure, and increases the risk for mortality. About 20% of patients do not recover to their pre-exacerbation state, leading to progressive decline [6]. Clinicians often rely on various tools and variables for assessing the severity and prognosis of an illness. This is important for carefully developing treatment plans and goals and providing truthful and reasonable counseling to patients and their families. Studies have demonstrated age, gender, body mass index, forced expiratory Volume_1_ (FEV_1_), cardiac reserve, nutritional status, arterial oxygen and carbon dioxide partial pressures, and C reactive protein (CRP) as predictors of mortality in AECOPD [7,8,9].

Several biomarkers have been studied as predictors of mortality in COPD. Many of these cannot be studied in low-resource settings in lower- and middle-income countries (LMICs). There is a need to identify simple biomarkers that could be used in primary care settings in LMICs. In this study, we try to identify whether simple, easily accessible hematological parameters can predict mortality in patients with acute exacerbation of COPD. Previous studies have attempted to explore whether hematological parameters can predict mortality. A study by River et al. [10] showed that low hemoglobin (anemia) can be predictive of mortality in AECOPD. Liška et al. [11] attempted to identify whether immature granulocytes (IG), activated neutrophils (NEUT, granularity intensity; NEUT, reactivity intensity), and lymphocytes (reactive LYMP; antibody synthesizing LYMP) could predict mortality; however, they were not able to identify a strong association. A study by Mendy et al. found that high CRP, neutrophilia, and eosinopenia could predict long-term mortality in AECOPD patients [12]. Studies have demonstrated that neutrophil lymphocyte ratio (NLR) can predict disease course in AECOPD [13].

## 2. Materials and Methods

### 2.1. Data Collection

This was a retrospective study conducted on patients with Acute Exacerbation of COPD admitted to a tertiary care teaching hospital in Mysuru, India. Ethical committee approval was obtained from the JSS Institutional Ethics Committee (Approval Number–JSSMC/IEC/090620/17NCT/2020-21) before commencing the project.

All adults (>18 years) who had been previously diagnosed with COPD, met criteria suggestive of an exacerbation, and were willing to give consent were included in the study. Patients managed on an outpatient basis, those who refused consent, or those with missing investigations were excluded from this study. 

The International Classification of Diseases (ICD) code J44.1 (Chronic Obstructive Pulmonary Disease with Acute Exacerbation–unspecified) was used to identify all records of patients admitted to the hospital from 1 January 2017 to 30 November 2022. A total of 2920 such records were identified. Given a 26.2% mortality rate seen in another study [14], a sample size of 298 was calculated at a 95% confidence level with a 5% margin of error. Systematic Random Sampling (computer generated) was used to identify 500 patients.

The Hospital Information System (HIS) was used to trace old records, reports, and summaries for these patients. The basic demographic details of the patients were entered into an MS Excel sheet. The investigations carried out at the time of admission were recorded and entered into the database. The hospital used the automated analyzer Sysmex XN1000 for analyzing samples. The hemoglobin, packed cell volume (PCV), red cell distribution width (RDW-CV), platelet count, reticulocyte count, total leukocyte count (TLC), absolute neutrophil count (ANC), lymphocyte count, urea, and creatinine were noted. Excel sheet formulas were created for calculating neutrophil lymphocyte ratio (NLR), platelet lymphocyte ratio (PLR), urea/creatinine (UC) ratio, and derived NLR (dNLR) (ANC/[TLC-ANC]).

### 2.2. Analysis

Means and standard deviations were calculated for age and all hematological parameters for patients who died and those who survived. The Shapiro–Wilk tests showed a significant departure from the normality when applied to the continuous variables. Pearson’s chi-square test was used for categorical variables while Student’s *t*-test was used for continuous variables. Receiver Operator Characteristics (ROC) curves were plotted. Youden’s J was used to identify ideal cut-off values for each of the parameters and sensitivity and specificity were calculated. Multivariate Cox regression analysis was then performed to find an independent association of the hematological parameters. Kaplan–Meir survival analysis was performed for NLR and dNLR. All statistical analyses were performed using SPSS software (v29).

## 3. Results

A total of 500 cases were enrolled and included in this study; 436 (87.2%) were male and 64 (12.8%) were female. The median age of the study population was 66.5 years. Of the 500 patients, 42 had died, giving a mortality rate of 8.4%. Chi-square test was used to identify the association between sex and mortality from acute exacerbation of Chronic Pulmonary Obstructive Disease (AECOPD) and this association was found to be insignificant. Student’s *t*-test was used to find the relationship between various hematological parameters such as TLC, ANC, NLR, dNLR, PLR, U/C ratio, hemoglobin, and PCV with mortality in AECOPD. The demographic and hematological variables for the study population are shown below in Table 1.

**Figure 1:**
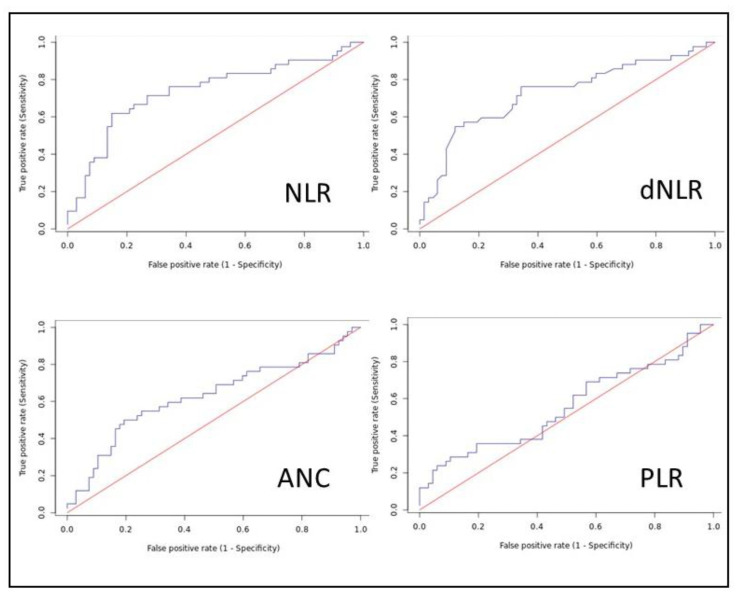
Receiver Operating Characteristics (ROC) curves.

The Receiver Operating Characteristics (ROC) curves were plotted for all hematological parameters with the student’s *t*-test showing *p* < 0.5, as seen in Figure 1 and Table 2. The areas under the curve (AUC) were obtained and were highest for the neutrophil/lymphocyte ratio (NLR), 0.737, followed by derived neutrophil/lymphocyte ratio (dNLR), 0.726. Youden’s J was used to identify ideal cut-off points and the sensitivity and specificity were calculated for that cut-off. The AUC and cut-off for the various variables are shown in Table 3.

### 3.1. Neutrophil/Lymphocyte Ratio (NLR)

A higher NLR was found to be significantly associated with mortality in patients with AECOPD. A Student’s *t*-test showed a statistically significant difference in the NLR of AECOPD patients who died (19.9 ± 16.9) vs. those who survived (9.67 ± 7.67). The AUC was 0.737 and the Youden’s J identified 14.839 as an ideal cut-off with a sensitivity of 61.9% and specificity of 85.1%. NLR was found to be an independent predictor of mortality in AECOPD.

### 3.2. Derived Neutrophil/Lymphocyte Ratio (dNLR)

A higher dNLR was found to be significantly associated with mortality in patients with AECOPD. A Student’s *t*-test showed a statistically significant difference in the dNLR of AECOPD patients who died (10.36 ± 6.53) vs. those who survived (5.94 ± 3.75). The AUC was 0.726 and the Youden’s J identified 9.38 as an ideal cut-off with a sensitivity of 54.8% and specificity of 88.1%.

### 3.3. Platelet/Lymphocyte Ratio (PLR)

A higher PLR was found to be significantly associated with mortality in patients with AECOPD. A Student’s *t*-test showed a statistically significant difference in the PLR of AECOPD patients who died (383.2 ± 392.6) vs. those who survived (270.5 ± 155.5). The AUC was 0.556 and the Youden’s J identified 451.32 as an ideal cut-off with a sensitivity of 28.6% and specificity of 89.6%. PLR was not found to be an independent predictor of mortality in AECOPD.

### 3.4. Red Cell Distribution Width (RDW)

The RDW was not found to be significantly associated with mortality in patients with AECOPD. A Student’s *t*-test showed a statistically insignificant difference in the RDW of AECOPD patients who died (15.11 ± 2.42) vs. those who survived (14.89 ± 2.49).

### 3.5. Total Leukocyte Count (TLC)

A higher TLC was found to be significantly associated with mortality in patients with AECOPD. A Student’s *t*-test showed a statistically significant difference in the TLC of AECOPD patients who died (14,225 ± 7869) vs. those who survived (11,577 ± 4896). The AUC was 0.608 and the Youden’s J identified 13,640 cells/mm^3^ as an ideal cut-off with a sensitivity of 54.8% and specificity of 76.1%. TLC was not found to be an independent predictor of mortality in AECOPD.

### 3.6. Absolute Neutrophil Count (ANC)

A higher ANC was found to be significantly associated with mortality in patients with AECOPD. A Student’s *t*-test showed a statistically significant difference in the ANC of AECOPD patients who died (12,655 ± 7499) vs. those who survived (9541 ± 4682). The AUC was 0.608 and the Youden’s J identified 12,556 cells/mm^3^ as an ideal cut-off with a sensitivity of 50% and specificity of 80.6%. ANC was not found to be an independent predictor of mortality in AECOPD.

### 3.7. Hemoglobin (Hb)

A lower Hb was found to be significantly associated with mortality in patients with AECOPD. A Student’s *t*-test showed a statistically significant difference in the Hb of AECOPD patients who died (12.66 ± 2.43) vs. those who survived (13.5 ± 1.96). The AUC was 0.594 and the Youden’s J identified 11.8 mg/dL as an ideal cut-off with a sensitivity of 86.6% and specificity of 40.5%. Hb was not found to be an independent predictor of mortality in AECOPD.

### 3.8. Packed Cell Volume (PCV)

A lower PCV was found to be significantly associated with mortality in patients with AECOPD. A Student’s *t*-test showed a statistically significant difference in the PCV of AECOPD patients who died (39.43 ± 8.01) vs. those who survived (42.48 ± 6.57). The AUC was 0.602 and the Youden’s J identified 36.6% as an ideal cut-off with a sensitivity of 86.6% and specificity of 42.9%. PCV was not found to be an independent predictor of mortality in AECOPD.

### 3.9. Urea/Creatinine Ratio (U/C)

The U/C was not found to be significantly associated with mortality in patients with AECOPD. A Student’s *t*-test showed a statistically insignificant difference in the U/C of AECOPD patients who died (37.5 ± 14.46) vs. those who survived (39.12 ± 14.92).

**Figure 2:**
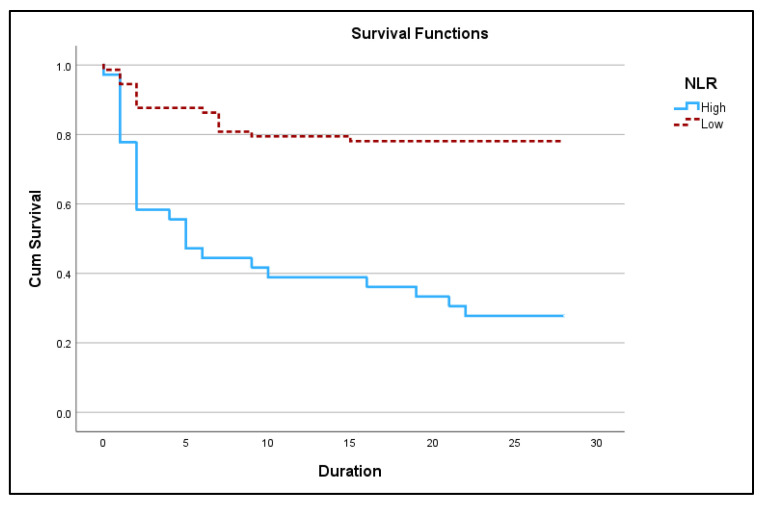
Kaplan–Meir 28-day survival plot for high NLR (≥14.83) vs. low NLR (<14.83) in acute exacerbation of COPD (*p <* 0.05).

**Figure 3:**
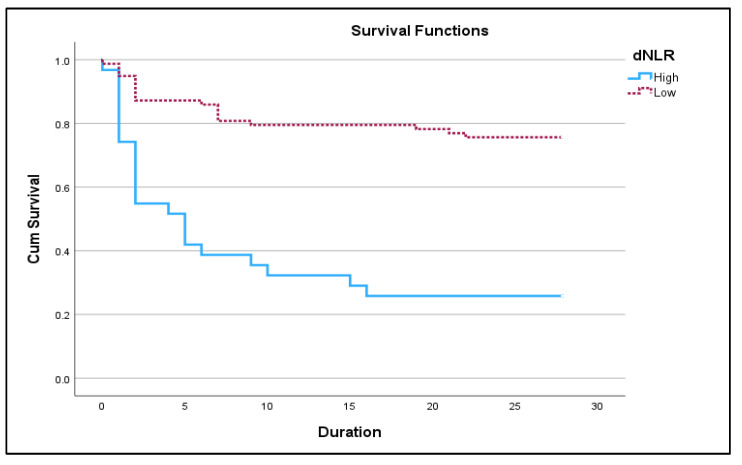
Kaplan–Meir 28-day survival plot for high dNLR (≥9.98) vs. low dNLR (<9.98) in acute exacerbation of COPD (*p <* 0.05).

Figure 2 and Figure 3 below show Kaplan Meir 28-day survival plots for NLR and dNLR respectively. 

## 4. Discussion

Acute exacerbation of COPD is a common complication of COPD and is one of the most common causes of rehospitalization in these patients. Repeat exacerbations lead to a progressive decline in the health of these patients and give rise to devastating complications that include but are not limited to respiratory failure, cor pulmonale, and even death. In our study, we found a 28-day mortality rate of 8.4%. Other studies have also found similar mortality rates. A meta-analysis by Hoogendoorn et al. [9] reported in-hospital mortality in exacerbation of COPD ranging from 2.5–14%, with ICU patients having a higher mortality rate of up to 30%. A study by Cao et al. [8] reported a mortality rate of 11.5% among patients in the respiratory intensive care unit.

In this study, we analyzed various hematological parameters such as NLR, TLC, dNLR, and ANC to identify whether these variables could predict mortality in patients with AECOPD. We found that NLR was an independent predictor of mortality in acute exacerbation of COPD. TLC, PLR, and ANC were found to be significantly associated in univariate Cox regression analysis but not in multivariate analysis. Among the demographic variables, age was an independent risk factor for mortality but not sex. The strongest relationship was seen with the neutrophil/lymphocyte ratio (NLR). A higher NLR was found to be significantly associated with mortality. Youden’s J identified 14.839 as an ideal cut-off with a sensitivity of 61.9% and specificity of 85.1%.

The neutrophil/lymphocyte ratio, which is a ratio of the absolute counts of these two leukocytes, was first established as a potential parameter of severe illness approximately two decades ago [15]. A pro-inflammatory state within the body promotes neutrophilia and lymphopenia [16]. This leads to an increase in the ratio. Therefore, the ratio and absolute count can be used to gauge the severity of the inflammation/infection within the body, which is likely how it becomes a marker for predicting mortality in these patients. This can be seen in our case where the absolute neutrophil count was also significantly higher in patients who died (12,655 ± 7499) vs. those who survived (9541 ± 4682). As seen in Table 4, the NLR cut-off has varied from study to study. We also found the cut-off in our study to be significantly higher compared to other studies.

This is not the first time that the NLR ratio has been used to predict mortality in an illness. For example, it has been demonstrated to be able to do so in COVID-19 [17,18]. NLR has also been found to be predictive of mortality in several chronic illnesses such as metastatic cancer, chronic liver disease, and psychiatric illnesses such as depression [19].

Platelets are also well-recognized inflammatory markers and they rise in response to inflammation [20]. Therefore, it is understandable that the platelet lymphocyte ratio was higher in patients who died (383.2 ± 392.6) vs. those who survived (270.5 ± 155.5). Platelet count as an independent marker of disease severity has been studied in illnesses such as COVID-19 and pneumonia [21,22]. PLR has also been used as a predictor of mortality in COPD [23]. However, in our study, although there was a significant difference in the PLR of patients who survived compared to those who did not, PLR was not able to predict mortality.

**Table 4:** Comparison of studies finding NLR as an independent predictor of mortality in AECOPD.

Author	Country	Number of Patients	Mean Age (Years)	NLR Cut-off	Odds Ratio
Present study	India	500	66.50	14.83	1.14
Duman et al. [24]	Turkey	1704	70.80	7.00	1.79
Xiong et al. [25]	China	368	70.60	3.30	3.58
Teng et al. [26]	China	906	81.86	10.35	1.06
Rahimirad et al. [27]	Iran	315	70.10	4.00	3.5
Yao et al. [28]	China	303	61.00	6.24	-
Luo et al. [23]	China	533	75.71	6.74	3.87
Karauda et al. [29]	Poland	275	69.42	13.2	-
Saltürk et al. [30]	Turkey	585	69.00	16	1.96
Ardestani et al. [31]	Iran	829	68.30	6.9	1.07
Liu et al. [32]	China	622	75.10 *	4.19	2.05
Yilmaz et al. [33]	Turkey	171	69.00 *	3.18	1.90
Esmaeel et al. [34]	Egypt	80	61.00	3.4	1.2
Lu et al. [35]	China	604	77.00	10.23	41.85
Bilir et al. [36]	Turkey	186	56.90	3.35	-

* Mean age of mortality group only.

We also see that patients who died had a lower Hb and PCV level than those who survived. Low levels of Hb and PCV had the highest sensitivity compared to all parameters assessed in this study (86.6% at a cut-off of 11.8 mg/dL for Hb and 86.6% at a cut-off of 36.6% for PCV). This is consistent with studies that have earlier demonstrated that lower hemoglobin levels are associated with higher all-time, all-cause mortality [37]. Hemoglobin has also been used for predicting mortality in patients with sepsis [38]. While this relationship is statistically significant, it is unlikely of any clinical importance due to the small difference compared to patients who survived (12.66 vs. 13.5). We also did not find Hb and PCV as predictors of mortality in our study. Red cell distribution width (RDW) has been used before in studies of cardiac disease and thromboembolism. Seyhan et al. [39] found RDW to be predictive of mortality in stable COPD patients; however, in our study, we did not find it to be a predictor of mortality. There was also no statistically significant difference in the geometric means of RDW between the survivors and those who died.

These findings are immensely helpful as complete blood count (CBC) is a routine, cheap, and easy-to-perform investigation, which is now ubiquitous in clinical practice, even in remote areas. However, it cannot be overstated that none of these parameters can alone predict mortality with a high degree of certainty. The highest sensitivity was for NLR, which was also just around 62%. While it was beyond the scope of this study, it is critical to also take into consideration the various comorbidities a patient may be suffering from. A study by Parthasarathi [40] has shown in a study of COVID-19 patients that comorbidities can influence and change the ideal cut-off of a hematological parameter for predicting mortality.

In this study, we studied 500 patients and assessed several hematological parameters that can be used in low-resource settings and therefore are cheap and easy to utilize. This study had a few limitations, as it was retrospective. The investigations at time of admission were considered and these values might have undergone considerable change by the time of death/discharge. The comorbidities of the patient were not taken into account in this analysis. A multi-center study with a larger sample size may be able to identify more variables that can predict mortality and may help further refine the results seen in this study, particularly with regard to identifying a better-suited cut-off.

## 5. Conclusions

In conclusion, we can say that in patients with acute exacerbation of COPD, NLR and age were predictors of mortality. There was a significant association between higher dNLR, PLR, ANC, and TLC, and lower Hb and PCV with mortality, even though these were not predictors of mortality.

## Figures and Tables

**Table 1:** Demographic and hematological parameters of the study populations.

Variable	OUTCOME		*p* Value
	Survived (*n* = 458)	Death (*n* = 42)	
Age (years)	65.94 ± 11.03	72.86 ± 9.09	<0.001 *
Sex			0.85 ^#^
Female (*n* = 64)	59	5	
Male (*n* = 436)	399	37	
Total Leukocyte Count (TLC) [cells/mm^3^]	11,577 ± 4896	14,225 ± 7869	0.017 *
Absolute Neutrophil Count (ANC) [cells/mm^3^]	9541 ± 4682	12,655 ± 7499	0.004 *
Neutrophil/Lymphocyte Ratio (NLR)	9.67 ± 7.67	19.9 ± 16.9	<0.001 *
Platelet/Lymphocyte Ratio (PLR)	270.5 ± 155.5	383.2 ± 392.6	0.01 *
Derived NLR (dNLR)	5.94 ± 3.75	10.36 ± 6.53	<0.001 *
Urea/Creatinine Ratio (U/C)	39.12 ± 14.92	37.5 ± 14.46	0.32 *
Hemoglobin [mg/dL] (Hb)	13.5 ± 1.96	12.66 ± 2.43	0.027 *
Packed Cell Volume (PCV) [%]	42.48 ± 6.57	39.43 ± 8.01	0.017 *
Red Cell Distribution Width (RDW) [%]	14.89 ± 2.49	15.11 ± 2.42	0.322 *

* Student’s *t* test, ^#^ Chi-square test.

**Table 2:** Univariable and multivariable Cox regression analysis of variables associated with AECOPD mortality.

Variable	Univariate		Multivariate	
	OR (95% CI)	*p* Value	OR (95% CI)	*p* Value
Age	1.05 (1.02–1.09)	<0.001	1.04 (1.00–1.07)	0.02
NLR	1.04 (1.02–1.05)	<0.001	1.05 (1.00–1.10)	0.02
ANC	1.00 (1.00–1.00)	<0.001	1.00 (1.00–1.00)	0.56
TLC	1.00 (1.00–1.00)	0.01	1.00 (1.00–1.00)	0.59
PLR	1.00 (1.00–1.00)	<0.001	0.99 (0.99–1.00)	0.22
Hb	0.89 (0.78–1.02)	0.09	0.93 (0.63–1.38)	0.74
PCV	0.96 (0.92–1.00)	0.07	0.99 (0.88–1.12)	0.95

**Table 3:** Area Under Curve (AUC), optimal cut-off values, sensitivity/specificity, and Likelihood Ratio (LR) for that cut-off.

Variable	AUC * (95% Ci)	Cut-Off ^#^	Sensitivity	Specificity	Likelihood Ratio	
					+	−
TLC	0.608 (0.492–0.723)	13,640	54.8%	76.1%	2.29	0.59
ANC	0.628 (0.514–0.742)	12,556	50.0%	80.6%	2.58	0.62
NLR	0.737 (0.635–0.84)	14.839	61.9%	85.1%	4.15	0.45
dNLR	0.726 (0.623–0.829)	9.9890	54.8%	88.1%	4.61	0.51
Hb	0.594 (0.477–0.71)	11.8	86.6%	40.5%	1.45	0.35
PCV	0.602 (0.485–0.72)	36.6	86.6%	42.9%	1.51	0.33
PLR	0.556 (0.44–0.673)	451.32	28.6%	89.6%	2.69	0.8

* From Receiver Operating Characteristics (ROC) curves with 95% Confidence Intervals in brackets, ^#^ Youden’s J is used for calculating optimal cut-off. CI–Confidence Interval.

## Data Availability

Data may be available upon request after appropriate blinding of patient identifying information.
